# A Bayesian taxonomic classification method for 16S rRNA gene sequences with improved species-level accuracy

**DOI:** 10.1186/s12859-017-1670-4

**Published:** 2017-05-10

**Authors:** Xiang Gao, Huaiying Lin, Kashi Revanna, Qunfeng Dong

**Affiliations:** 1 Department of Public Health Sciences, Loyola University Chicago Health Sciences Division, Maywood, IL 60153 USA; 2 Center for Biomedical Informatics, Loyola University Chicago Health Sciences Division, Maywood, IL 60153 USA; 3 Bioinformatics Program, Loyola University Chicago Lake Shore Campus, Chicago, IL 60660 USA; 4 Department of Computer Science, Loyola University Chicago Water Tower Campus, Chicago, IL 60611 USA

**Keywords:** 16S rRNA gene, Taxonomic classification

## Abstract

**Background:**

Species-level classification for 16S rRNA gene sequences remains a serious challenge for microbiome researchers, because existing taxonomic classification tools for 16S rRNA gene sequences either do not provide species-level classification, or their classification results are unreliable. The unreliable results are due to the limitations in the existing methods which either lack solid probabilistic-based criteria to evaluate the confidence of their taxonomic assignments, or use nucleotide k-mer frequency as the proxy for sequence similarity measurement.

**Results:**

We have developed a method that shows significantly improved species-level classification results over existing methods. Our method calculates true sequence similarity between query sequences and database hits using pairwise sequence alignment. Taxonomic classifications are assigned from the species to the phylum levels based on the lowest common ancestors of multiple database hits for each query sequence, and further classification reliabilities are evaluated by bootstrap confidence scores. The novelty of our method is that the contribution of each database hit to the taxonomic assignment of the query sequence is weighted by a Bayesian posterior probability based upon the degree of sequence similarity of the database hit to the query sequence. Our method does not need any training datasets specific for different taxonomic groups. Instead only a reference database is required for aligning to the query sequences, making our method easily applicable for different regions of the 16S rRNA gene or other phylogenetic marker genes.

**Conclusions:**

Reliable species-level classification for 16S rRNA or other phylogenetic marker genes is critical for microbiome research. Our software shows significantly higher classification accuracy than the existing tools and we provide probabilistic-based confidence scores to evaluate the reliability of our taxonomic classification assignments based on multiple database matches to query sequences. Despite its higher computational costs, our method is still suitable for analyzing large-scale microbiome datasets for practical purposes. Furthermore, our method can be applied for taxonomic classification of any phylogenetic marker gene sequences. Our software, called BLCA, is freely available at https://github.com/qunfengdong/BLCA.

## Background

High-throughput 16S rRNA gene sequencing is widely used in microbiome studies for characterizing bacterial community compositions. A key computational task is to perform taxonomic classification for 16S rRNA gene sequences, with emphasis increasing on species-level classification [1]. The published tools dedicated for 16S rRNA gene classification include the RDP Classifier [2], 16S Classifier [3] and SPINGO [4]. There are also software packages or websites that provide 16S classification options, e.g., QIIME [5] and MG-RAST [6].

Despite the availability of those taxonomic classification tools, species-level classification for 16S rRNA gene sequences still remains a serious challenge for microbiome researchers. Some of the tools simply do not classify at the species level. For example, the standard version of the widely-used software, RDP Classifier, only classifies 16S rRNA gene sequences from the phylum to genus levels, although the RDP Classifier can be re-trained for species level classification. Another recently published software, the 16S Classifier, is not capable of classifying sequences at the species level either. For the other tools that can classify at the species level, they suffer from at least one of the two major limitations: i) nucleotide k-mer frequency is used for measuring similarity between query and database sequences, a proxy measurement of true sequence similarity; ii) solid probabilistic-based criteria is lacking for evaluating the confidence of taxonomic assignment results, particularly to evaluate whether the best-matched database sequence is significantly better than other database matches for the taxonomic assignments.

Taxonomic classification of 16S gene sequences typically requires comparing query sequences to annotated database sequences. The k-mer based approaches, e.g., the RDP Classifier and SPINGO, compare the frequency of k-mer nucleotides between query and database sequences. The higher degree of shared k-mer nucleotide frequencies, the more similar the two sequences are. The advantage of k-mer based approaches is its fast computational speed. However, k-mer based approaches rely on two key assumptions: i) the k-mer nucleotides in DNA sequences used as discriminating features among different taxa are independent, and ii) the actual nucleotide position of the k-mers in the DNA sequences is not important. In reality, nucleotides in different positions of a gene sequence can be correlated (e.g., to preserve the secondary or higher-dimensional structure of rRNA folding), and gene sequences with the same set of k-mer in different orders are clearly not the same sequences. Therefore, these two assumptions are the theoretical sources of taxonomic misclassification by k-mer based approaches. There is also a nontrivial practical limitation for a k-mer based approach: it is extremely difficult to determine an optimal size of k-mer for discriminating among different species at different regions of 16S sequences. For example, the accuracy of the RDP Classifier, which uses a k-mer size of eight, varies significantly with different types of bacterial taxa at different 16S gene regions [7]. Therefore, k-mer based approaches rely on a proxy measurement of the sequence similarity between the query and database sequences, which is inherently less accurate than the gold standard sequence-alignment-based method.

As mentioned above, another major limitation for most existing methods is that they lack solid probabilistic-based criteria to evaluate the confidence of their taxonomic assignments. Although all existing methods infer taxonomic classification based on matched database sequences, most of the existing methods do not provide any indication on whether the best-matched database hit sequence is significantly better than other database hits. Since the 16S rRNA gene is highly conserved among different bacterial taxa and the query sequences in microbiome studies are often only a short fragment of the full-length 16S rRNA gene with sequencing errors, it is common to have several database hits from different taxa that may have comparable sequence similarities to the query sequence. Therefore, it is not reliable to simply transfer the taxonomic annotation associated with the best database hit for the query sequence [8]. Instead, a better method for 16S classification may consider multiple database hits together and evaluate whether the best database hit is significantly better than other database hits.

The Lowest Common Ancestor (LCA) algorithm, implemented in the MEGAN package [9], provides a natural biological framework to integrate taxonomic annotations associated with multiple database hits when classifying query sequences. In MEGAN, all taxa corresponding to the BLAST [10] hits are first mapped to NCBI taxonomic trees and the lowest common ancestor of all mapped taxa is then assigned to the query sequence. For example, if a query sequence has two BLAST hits belonging to two different species, e.g., one from *Lactobacillus acidophilus* and the other one from *L. casei*, the LCA algorithm assigns the query sequence to the genus *Lactobacillus*, which is the lowest common taxonomic level of these two species. However, the LCA algorithm fails to consider the differing degrees of similarity between the query and the database hit sequences. In other words, when inferring the LCA for the query, the algorithm acts as if all the hit sequences, affected by an arbitrary sequence similarity threshold, were equally similar to the query sequence, even though in practice they are often not. Biologically speaking, the greater the degree of sequence similarity between the query and the hit sequences, the more likely they may belong to the same taxon, but the current LCA algorithm lacks a quantitative way to incorporate this important information on sequence similarity in its taxonomic assignment.

To overcome the above limitations of the existing software, we have developed a Bayesian-based LCA method, named BLCA. BLCA can perform species and even sub-species level taxonomic classification. It relies on sequence alignment instead of k-mer frequency for sequence similarity measurement; it considers multiple database hits instead of only the best database hit for taxonomic assignment; it provides a probabilistic-based confidence score for evaluating taxonomic assignments. The novelty of our method is that the contribution of each database hit to the taxonomic assignment of the query sequence is weighted by a Bayesian posterior probability based upon the sequence similarity of the database hit to the query. The calculated Bayesian posterior probability implicitly penalizes dissimilar database hit sequences in a quantitative way, which makes our method insensitive to arbitrary sequence similarity thresholds for selecting candidate database hits for each query sequence. We show that BLCA provides significantly more accurate classification results at the species level when compared to all other existing tools.

## Implementation

**Fig. 1:**
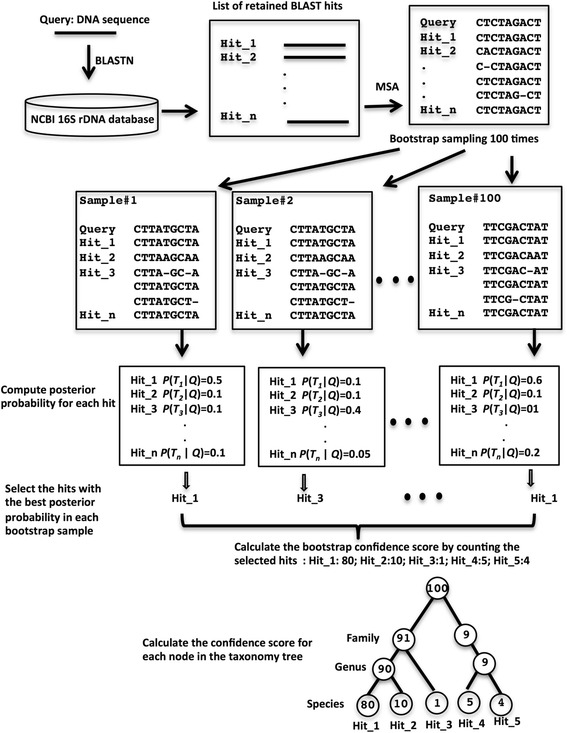
The overview of the BLCA algorithm. See main text for details

The BLCA method is implemented as a Python package, which is freely available at https://github.com/qunfengdong/BLCA under the GNU General Public License. An overview of the BLCA method is illustrated in Fig. 1. Users start by comparing the query 16S sequences against entries in an annotated 16S database using BLASTN. The taxonomic lineage of each 16S database sequence is extracted from the NCBI taxonomic database (ftp://ftp.ncbi.nih.gov/pub/taxonomy/). As with MEGAN, we chose the 16S rRNA gene collection from NCBI (ftp://ftp.ncbi.nlm.nih.gov/blast/db/16SMicrobial.tar.gz) as the default database, although users can also use the Greengenes database [11] or adopt any custom collection of 16S sequences provided that the sequence IDs can be mapped to the NCBI or Greengenes taxonomy. Next, the BLAST hits are extracted; by default, BLCA only extracts the BLAST hits from BLAST pairwise alignments with at least 95% identity and 95% coverage with respect to the query, but users can easily change these parameters using the command-line at execution as well as setting an additional criterion to retain only the BLAST hits whose bit scores are within a certain percentage of difference from the top hits (the same criterion used by MEGAN). Each query sequence and its corresponding BLAST hits are passed as an input to the MUSCLE program [12] for multiple sequence alignment. Because most 16S query sequences are not full-length gene sequences in practice, BLCA only extracts the relevant subsequences of the hits – those that actually align to the query sequences in the BLAST pairwise sequence alignments. An extra 10 nucleotides upstream and downstream relative to the aligned regions from the hit sequences are also included to avoid potential overhangs at the 5′ or 3′ end of the query sequences in the multiple sequence alignment.

We define *Pr*(*T*_*i*_ | *Q*) as the Bayesian posterior probability for a taxon *T*_*i*_ being assigned to a given query sequence *Q*. Based on Bayes’ rule, we obtain

1 $$ Pr\left({T}_i\left| Q\right.\right)= Pr\left( Q\left|{T}_i\right.\right) Pr\left({T}_i\right)/ Pr(Q) $$

wherein *Pr*(*Q* | *T*_*i*_) is the likelihood of observing the sequence Q if it were derived from the taxon *T*_*i*_. The likelihood can be calculated as the pairwise alignment score between the query sequence *Q* and the database hit sequence annotated as *T*_*i*_, divided by the pairwise alignment score between the hit sequence *T*_*i*_ to itself. In other words, the likelihood is defined as the similarity score between the query and the database hit normalized by the maximum possible similarity score between any sequences to the hit sequence. The likelihood *Pr*(*Q* | *T*_*i*_) is a real number between 0 (i.e., no match between the query *Q* and the database hit *T*_*i*_) and 1 (i.e., a perfect match between the query and the database hit). Our definition of *Pr*(*Q* | *T*_*i*_) as a likelihood simply reflects the degree of support by the evidence (i.e., similarity between query and the database hit) for the hypothesis (i.e. the query belongs to the taxon of the database hit). In our current implementation, the pairwise alignment score between the query sequence and BLAST hit sequence is computed from the multiple sequence alignment, which tends to be more accurate than the original BLAST pairwise alignment because BLAST alignment performs local alignment, whereas MUSCLE is a global alignment program. Since the alignment is between DNA sequences, the pairwise alignment score can be simply computed with the following criteria: match = +1, mismatch = −2, and gap = −2.5 (these are the exact default scoring criteria used for BLASTN). *Pr*(*T*_*i*_) is the prior probability of a particular taxon *T*_*i*_ for the query sequence, which is set to a uniform distribution in our implementation. The uniform prior is a suitable choice for taxonomic classification, since, without knowing the data, we can treat every taxon as equally probable (the same uniform prior is used in the RDP Classifier). If necessary, non-uniform priors can be easily adopted for specific situations where certain taxa are more likely than others in the same Bayesian framework described in this work. *Pr*(*Q*) is the marginal distribution of the query sequence *Q*, which can be calculated as the summation of the product of likelihoods and priors of all the BLAST hits, i.e., ∑_*i* = 1_^*m*^ *Pr*(*Q*|*T*_*i*_)*Pr*(*T*_*i*_) for *m* total BLAST hits, based on the law of total probability. Note that the term *Pr*(*T*_*i*_), assumed to be a uniform prior, can be cancelled from the denominator and numerator when calculating *Pr*(*T*_*i*_ | *Q*). In addition, sequence similarity estimations might be improved by specifying sequencing error models for both query and database sequences (e.g., a Poisson probability distribution of an observed nucleotide in a DNA sequence being incorrect); these can be incorporated in our Bayesian framework by adjusting the likelihood calculation in Eq. (1).

Since *T*_*i*_ corresponds to the taxonomic annotation for an individual BLAST hit sequence, it represents the leaf node in the NCBI taxonomic tree (e.g., at the species or sub-species level). We also need to compute the posterior probability at higher taxonomic levels, i.e., the internal nodes in the taxonomic tree that correspond to the antecedents of *T*_*i*_ (i.e., the common ancestors of all the *T*_*i*_). Using the addition rule for probability, the posterior probability of any internal node *I*, *Pr*(*T*_*I*_ | *Q*), in the taxonomic tree can be computed by a simple summation of those of all the descendant leaf *T*_*i*_:

2 $$ Pr\left({T}_I\left| Q\right.\right)={\displaystyle {\sum}_{i=1}^k \Pr \left({T}_i\left|{Q}_i\right.\right)} $$

wherein the internal node *I* has *k* total descendant leaf nodes. The Eq. (2) allows us to easily compute the posterior probability of any higher taxonomic level, e.g., from genus to phylum, by simply summing the posterior probabilities associated with all the descendant leaf nodes in the taxonomic trees under any internal nodes. Using the previous example in which a query sequence has one BLAST hit from *L. acidophilus* and the other from *L. casei*, the posterior probability for the genus level of *Lactobacillus* for the query is the sum of the posterior probabilities for *L. acidophilus* and *L. casei*, respectively.

Based on the posterior probabilities calculated for all the nodes in the taxonomic tree, a bootstrap confidence score is derived to evaluate the reliability of the taxonomic assignment for each node. Specifically, aligned nucleotide positions in the multiple sequence alignment between query and BLAST hits are randomly sampled with replacement; the total number of sampled nucleotide positions is the same as the length of the query sequence (i.e., a pseudo multiple-sequence alignment is bootstrapped from the original multiple-sequence alignment). Using the pseudo multiple-sequence alignment, the posterior probability of each leaf node in the taxonomic tree is re-computed by the same procedure as described above and the leaf node with the highest posterior probability is identified and tallied as the “winning” node. The process is repeated 100 times, and the number of times that a leaf node emerged as the winner becomes the confidence score for the taxonomic assignment of the particular node. Similar to the posterior probability calculation, the confidence score for internal nodes can also be obtained by summing up the confidence scores of all their descendent leaf nodes. The RDP Classifier uses a similar bootstrapping strategy to assign confidence scores for its taxonomic classifications. However, unlike the RDP Classifier, which is based on bootstrapping k-mers from query and database sequences, our strategy randomly samples from aligned nucleotides in multiple sequence alignment, a method that is commonly used for evaluating the confidence of branches in molecular phylogenic trees [13].

To assess the accuracy of a classification tool, we must have a benchmark dataset with known taxonomic annotations for each 16S sequence. Therefore, we extracted the V2, V4, V1–V3, V3–V5, and V6–V9 regions of 16S sequences from 1000 randomly selected bacterial species with known taxonomic annotations in the NCBI database as the benchmark dataset. These variable regions were chosen for testing because they represent typical 16S sequences in real-world microbial studies. Instead of using the exact sequences from those regions for testing, we introduced sequencing errors to each sequence, using a customized Python script to generate an average of 1% random mutation based on a Poisson distribution. The 1% mutation rate is based on the reported upper range of the Illumina MiSeq sequencing platform [14]. The test sequences, with sequencing errors, were searched against the 16S sequences from NCBI (downloaded on August 5th, 2016) using BLASTN version 2.5.0. For MEGAN parameters, we set the same default settings (e.g., minimum BLAST bit scores, maximum BLAST expected values, and the percent of BLAST hits) for both BLCA and MEGAN. For BLCA, SPINGO, and the RDP Classifier, two sets of confidence score thresholds were used: (i) 0.8–RDP Classifier’s default confidence score and (ii) 0.5–RDP Classifier’s confidence score threshold recommended for short-read sequences, as written in the RDP Classifier’s documentation. Neither MEGAN nor Kraken [15] have a probabilistic-based parameter for evaluating the assigned taxa, thus we used their default taxonomic assignments for comparison.

For each of the taxa in the benchmark dataset (e.g., a known *E. coli* sequence), we were able to identify whether the classification results from each software represent a true positive (TP, e.g., the predicted taxonomy is also *E. coli*), false negative (FN, e.g., the predicted taxonomy is not *E. coli*), false positive (FP, e.g., other non-*E. coli* sequences were incorrectly predicted to be *E. coli*), and true negative (TN, e.g., other non-*E. coli* sequences were correctly predicted to be non-*E. coli*). The total amount of TP, FN, FP, and TN are tallied from the 1000 test sequences from the species to the phylum levels. The rates of TP, FN, FP, and TN were used for computing the F-score, which is a standard measure of a classifier’s accuracy by combining both the precision and the recall of the classifier [16]. The procedure above was repeated three times to measure the variability of the classification accuracy.

Besides the above-simulated dataset, we also evaluated the performance of BLCA with a real-world 16S dataset, which was suggested by one of the reviewers of our manuscript. The dataset was originally produced by Pop et al. [17] and is available in the Bioconductor package (referred as the *msd16s* dataset) [18]. The *msd16s* dataset contains 26,044 species-level operational taxonomic unit (OTU) sequences from the V1V2 rRNA gene region. The original authors used the top BLAST hit against the RDP 16S database [19] as the taxonomic annotation for each OTU sequence. Since MEGAN and SPINGO can only use NCBI taxonomy nomenclature, we re-annotated the *msd16s* dataset by using the top BLAST hit against NCBI 16S database (i.e., the same BLAST strategy as in the original study of Pop et al. [17]) in order to ensure that MEGAN and SPINGO can be compared against BLCA and other programs using the same reference taxonomic annotation.

## Results

To compare BLCA against other software, we reviewed all recently published 16S taxonomic classification tools. Since BLCA aims to improve species-level classification accuracy compared to existing tools, we excluded the 16S Classifier program since it cannot classify at the species level.

To obtain a fair comparison with MEGAN (version 6.7.1), we used the same default criteria as MEGAN for retaining the BLAST hits. The most important MEGAN parameter for extracting BLAST hits for downstream analysis is the parameter *topPercent*, used to keep only the BLAST hits whose bit scores are within a given percentage of the best BLAST hit. The default value in MEGAN for this parameter is 10%. For example, if the top BLAST hit has a bit score of 1000, we only retain BLAST hits for downstream analysis if their BLAST bit scores are at least 900 (i.e., 1000–1000*10%). As shown in Table 1, BLCA consistently outperforms MEGAN with all the tested 16S variable regions from the species to the family levels of taxonomic classification. From the order to the phylum levels, the accuracies of BLCA, MEGAN and other software are similar and above 98% (data not shown). More importantly, the accuracy of MEGAN drops significantly when the *topPercent* filter was relaxed from 5 to 10% and further to 20% (the recommended range by the original MEGAN publication) at both the species and genus levels (Table 2). For example, using V1–V3 sequences, the species-level accuracy of MEGAN, measured by the F-scores, drops from 0.8394 (with *topPercent* set to 5%) to 0.7071 (with *topPercent* set to 10%), and further down to 0.4673 (with *topPercent* set to 20%). Besides V1–V3, these same trends are observed for all other tested 16S regions (Table 2). These results are expected because, by relaxing this parameter, more dissimilar BLAST hits (i.e., potentially “bad” BLAST hits) are included in the analysis and the inclusion of bad BLAST hits leads to erroneous taxonomic assignments. This reveals a fundamental limitation of the MEGAN method: its results are sensitive to which BLAST hits are included for analysis and it lacks a probabilistic method to penalize bad BLAST hits. Conversely, the results from BLCA, which showed higher accuracy than MEGAN, remained robust to the number of included BLAST hits (Table 2) since bad BLAST hits are penalized using posterior probability scores assigned by the BLCA algorithm. It is worth noting that it is unrealistic to prevent the inclusion of bad BLAST hits in a typical large-scale data analysis since there is no universal cutoff to exclude bad BLAST hits. Any such cutoffs are heuristic in nature, as such, they are inevitably either too stringent or not stringent enough.

**Table 1:** Comparison of the classification accuracies using the simulated dataset

CST = 0.8		V2	V4	V1V3	V3V5	V6V9
Species	BLCA	0.7594 ± 0.0164*	0.5331 ± 0.0208	0.9323 ± 0.0054*	0.8335 ± 0.0072*	0.8690 ± 0.0012*
	Kraken	0.7275 ± 0.0054	0.5326 ± 0.0181	0.8672 ± 0.0072	0.7542 ± 0.0087	0.7572 ± 0.0056
	MEGAN	0.7290 ± 0.0114	0.5238 ± 0.0161	0.7071 ± 0.0053	0.5206 ± 0.0108	0.5227 ± 0.0140
	RDP	0.6102 ± 0.0042	0.3928 ± 0.0292	0.8549 ± 0.0199	0.7307 ± 0.0203	0.7823 ± 0.0124
	SPINGO	0.5700 ± 0.0187	0.3910 ± 0.0106	0.7907 ± 0.0061	0.6900 ± 0.0071	0.7318 ± 0.0116
Genus	BLCA	0.9498 ± 0.0019*	0.8982 ± 0.0107*	0.9965 ± 0.0012*	0.9863 ± 0.0011*	0.9925 ± 0.0012*
	Kraken	0.9072 ± 0.0066	0.8612 ± 0.0189	0.9691 ± 0.0051	0.9463 ± 0.0006	0.9437 ± 0.0034
	MEGAN	0.9334 ± 0.0079	0.8830 ± 0.0115	0.9528 ± 0.0040	0.9002 ± 0.0027	0.8939 ± 0.0041
	RDP	0.8768 ± 0.0065	0.8067 ± 0.0139	0.9629 ± 0.0072	0.9562 ± 0.0065	0.9657 ± 0.0042
	SPINGO	0.8481 ± 0.0002	0.7726 ± 0.0077	0.9333 ± 0.0057	0.9192 ± 0.0034	0.9238 ± 0.0067
Family	BLCA	0.9791 ± 0.0009*	0.9787 ± 0.0018*	0.9984 ± 0.0019*	0.9975 ± 0.0019*	0.9970 ± 0.0014*
	Kraken	0.9594 ± 0.0038	0.9480 ± 0.0028	0.9882 ± 0.0021	0.9850 ± 0.0033	0.9799 ± 0.0032
	MEGAN	0.9495 ± 0.0089	0.9413 ± 0.0015	0.9517 ± 0.0032	0.9397 ± 0.0044	0.9447 ± 0.0034
	RDP	0.9461 ± 0.0093	0.9295 ± 0.0062	0.9818 ± 0.0007	0.9806 ± 0.0054	0.9855 ± 0.0013
	SPINGO	NA	NA	NA	NA	NA
CST = 0.5		V2	V4	V1V3	V3V5	V6V9
Species	BLCA	0.8485 ± 0.0128*	0.6813 ± 0.0115*	0.9629 ± 0.0077*	0.9050 ± 0.0034*	0.9315 ± 0.0045*
	Kraken	0.7275 ± 0.0054	0.5326 ± 0.0181	0.8672 ± 0.0072	0.7542 ± 0.0087	0.7572 ± 0.0056
	MEGAN	0.7290 ± 0.0114	0.5238 ± 0.0161	0.7071 ± 0.0053	0.5206 ± 0.0108	0.5227 ± 0.0140
	RDP	0.7526 ± 0.0107	0.5692 ± 0.0194	0.8997 ± 0.0144	0.8221 ± 0.0105	0.8621 ± 0.0094
	SPINGO	0.6570 ± 0.0124	0.5008 ± 0.0114	0.8256 ± 0.0038	0.7497 ± 0.0041	0.7805 ± 0.0021
Genus	BLCA	0.9722 ± 0.0028*	0.9467 ± 0.0031*	0.9985 ± 0.0019*	0.9947 ± 0.0013*	0.9972 ± 0.0002*
	Kraken	0.9072 ± 0.0066	0.8612 ± 0.0189	0.9691 ± 0.0051	0.9463 ± 0.0006	0.9437 ± 0.0034
	MEGAN	0.9334 ± 0.0079	0.8830 ± 0.0115	0.9528 ± 0.0040	0.9002 ± 0.0027	0.8939 ± 0.0041
	RDP	0.9319 ± 0.0044	0.8960 ± 0.0086	0.9710 ± 0.0049	0.9693 ± 0.0046	0.9729 ± 0.0003
	SPINGO	0.8807 ± 0.0034	0.8354 ± 0.0041	0.9400 ± 0.0030	0.9287 ± 0.0024	0.9317 ± 0.0083
Family	BLCA	0.9870 ± 0.0013*	0.9856 ± 0.0035*	0.9987 ± 0.0021*	0.9991 ± 0.0012*	0.9984 ± 0.0019*
	Kraken	0.9594 ± 0.0038	0.9480 ± 0.0028	0.9882 ± 0.0021	0.9850 ± 0.0033	0.9799 ± 0.0032
	MEGAN	0.9495 ± 0.0089	0.9413 ± 0.0015	0.9517 ± 0.0032	0.9397 ± 0.0044	0.9447 ± 0.0034
	RDP	0.9696 ± 0.0040	0.9674 ± 0.0015	0.9836 ± 0.0017	0.9830 ± 0.0033	0.9868 ± 0.0004
	SPINGO	NA	NA	NA	NA	NA

Each entry in the table shows the average and standard deviation of the F-scores for a particular classifier (i.e., rows) at a specific 16S region (i.e., columns) based on three random sets of 1000 test sequences. Two confidence score thresholds (CST), 0.8 and 0.5, were applied for BLCA, RDP Classifier, and SPINGO as described in the main text. The *indicates that the F-scores of BLCA are significantly higher than those of other software, based on a one-tailed paired *t*-test with a *p*-value less than 0.05. Similar statistical significance was also obtained using the one-tailed Wilcoxon signed-rank test. Note that the SPINGO program does not produce family-level classification. In addition, Kraken and MEGAN do not provide any probabilistic-based parameters for evaluating the assigned taxa, thus we used their default taxonomic assignments for comparison

**Table 2:** BLCA accuracy is insenesitve to the inclusion of dissimilar BLAST hits

Taxonomic levels		Genus		Species	
16S region	*topPercent* Filter	BLCA	MEGAN	BLCA	MEGAN
V2	5%	0.9539 ± 0.0038	0.9531 ± 0.0044	0.7747 ± 0.0150	0.8091 ± 0.0153
	10%	0.9498 ± 0.0019	0.9334 ± 0.0079	0.7594 ± 0.0164	0.7290 ± 0.0114
	20%	0.9487 ± 0.0018	0.8966 ± 0.0080	0.7580 ± 0.0176	0.5983 ± 0.0075
V4	5%	0.9078 ± 0.0078	0.9230 ± 0.0082	0.5597 ± 0.0175	0.6497 ± 0.0058
	10%	0.8982 ± 0.0107	0.8830 ± 0.0115	0.5331 ± 0.0208	0.5238 ± 0.0161
	20%	0.8965 ± 0.0092	0.8016 ± 0.0041	0.5317 ± 0.0189	0.3915 ± 0.0119
V1V3	5%	0.9960 ± 0.0009	0.9778 ± 0.0006	0.9314 ± 0.0058	0.8394 ± 0.0069
	10%	0.9965 ± 0.0012	0.9528 ± 0.004	0.9323 ± 0.0054	0.7071 ± 0.0053
	20%	0.9959 ± 0.0009	0.8609 ± 0.0087	0.9321 ± 0.0053	0.4673 ± 0.0150
V3V5	5%	0.9865 ± 0.0020	0.9550 ± 0.0041	0.8380 ± 0.0064	0.7025 ± 0.0112
	10%	0.9863 ± 0.0011	0.9002 ± 0.0027	0.8335 ± 0.0072	0.5206 ± 0.0108
	20%	0.9863 ± 0.0011	0.7369 ± 0.0094	0.8361 ± 0.0039	0.2880 ± 0.0061
V6V9	5%	0.9933 ± 0.0011	0.9532 ± 0.0050	0.8722 ± 0.0066	0.7258 ± 0.0129
	10%	0.9925 ± 0.0012	0.8939 ± 0.0041	0.8690 ± 0.0012	0.5227 ± 0.0140
	20%	0.9931 ± 0.0017	0.7138 ± 0.0083	0.8701 ± 0.0050	0.2691 ± 0.0255

The parameter *topPercent* is for keeping only the BLAST hits whose bit scores are within a given percentage of the best BLAST hit. The larger the parameter is, the more dissimilar database hits are included for taxonomic classification for the query sequence. The default value in MEGAN for this parameter is 10%. In our comparisons, we set the value of *topPercent* to be 5, 10 and 20% for both BLCA and MEGAN, the recommended range by the original MEGAN publication, to compare the performance of BLCA and MEGAN under different stringencies of retaining BLAST hits. Each table entry shows the average and standard deviation of the F-scores, based on the confidence score threshold of 0.8, for each tested software at the corresponding 16S region. The F-scores of BLCA are much less sensitive to the value of *topPercent* when compared to MEGAN

The SPINGO program is specifically designed for species-level classification. The authors of SPINGO even showed that SPINGO has superior classification accuracy compared to a customized RDP Classifier and best-matched BLAST hits at species level [4]. Like BLCA and MEGAN, SPINGO uses the NCBI taxonomic database for taxonomic assignments. Unlike those tools, however, SPINGO uses a k-mer based approach instead of sequence alignment to measure the similarity between query and database sequences. The only threshold for SPINGO is its confidence score for taxonomic assignments, which is compatible with the BLCA confidence score. Table 1 shows that the accuracy of BLCA is statistically significantly higher than that of SPINGO in all tested 16S regions at the confidence score thresholds of 0.8 and 0.5, respectively. In addition, SPINGO cannot do subspecies classification, nor can it do family or higher level classification, whereas BLCA can classify reads from any level ranging from subspecies to phylum (though there are not enough annotated subspecies datasets at NCBI for evaluating BLCA subspecies-level classification accuracy).

Even though the standard release of the RDP Classifier cannot classify 16S sequences at the species level, we obtained the training script from the RDP Classifier’s development team (personal communications) and re-trained the RDP Classifier for species-level classification with the same NCBI 16S database that BLCA uses. The NCBI 16S database is used because MEGAN and SPINGO must use NCBI taxonomic database. Therefore, the NCBI database provides a common ground for evaluating the results of all of these tools on the basis of their computational algorithms without being influenced by different taxonomic standards. Similar to SPINGO, the RDP Classifier’s confidence score is also compatible with the BLCA confidence score. Although the default threshold for the RDP Classifier’s confidence score is 0.8, the developers of the RDP Classifier also recommend a threshold of 0.5 for short read classification. Our results show that BLCA has higher accuracy than the RDP Classifier at the thresholds of 0.8 and 0.5 (Table 1).

Besides these 16S-specific classification tools, there are also metagenomic classification tools that are designed for identifying microbial taxa from whole metagenome shotgun (WMS) sequences. We have chosen Kraken [15] as a representative WMS classification tool to compare with BLCA. Kraken is chosen because of two reasons: i) it has superior or comparable classification accuracy to other existing WMS tools [20] and ii) to our best knowledge, it is the only WMS tool that has been successfully applied in a published 16S study [21]. Kraken’s default database incorporates reference genome sequences. To have a fair comparison with BLCA, we have replaced Kraken’s default database with the same NCBI 16S database used for BLCA, thus increasing its sensitivity to classify a broader range of bacterial taxa. Kraken, a k-mer based program seeking best database matches, does not provide any confidence score to evaluate the confidence of assigned taxonomies, although Kraken’s output can be filtered based on the percent of k-mers matched to each taxa (no guidance is provided by its developer on how to set the filtering threshold). As shown in Table 1, even allowing the maximum sensitivity for Kraken (i.e., without any filtering of Kraken’s output), which is the default setting for Kraken, BLCA still significantly outperforms Kraken with all tested 16S regions from the species to the family level.

In addition to using simulated datasets to evaluate BLCA and other software, Table 3 shows that BLCA had either higher or comparable classification accuracies when tested with a real-world 16S dataset. For example, with a confidence score threshold of 0.5 (the recommended threshold for the RDP Classifier for short sequence reads), the species-level classification accuracy of BLCA, measured using an F-score, is 0.716, much higher than the classification accuracy of MEGAN (0.544), the RDP Classifier (0.613), and SPINGO (0.562). The same trends were observed when the default confidence score threshold of 0.8 was applied (Table 3). It is worth noting that, as this is a real-world dataset, the true taxonomic classification is unknown. We had to rely on the top BLAST hit as the reference taxonomic classification when we evaluated the classification accuracies of each software. Nonetheless, the results from the real-world dataset were consistent with those from the simulated datasets, showing that BLCA tends to produce higher taxonomic classification accuracies than currently existing software.

**Table 3:** Comparison of the classification accuracies using a real-world dataset

Taxonomy Level	Method	V1V2 Region	
		CST = 0.8	CST = 0.5
Species	BLCA	0.570	0.716
	Kraken	0.589	0.589
	MEGAN	0.544	0.544
	RDP	0.490	0.613
	SPINGO	0.486	0.562
Genus	BLCA	0.729	0.79
	Kraken	0.694	0.694
	MEGAN	0.745	0.745
	RDP	0.643	0.708
	SPINGO	0.605	0.650
Family	BLCA	0.814	0.832
	Kraken	0.777	0.777
	MEGAN	0.869	0.869
	RDP	0.775	0.805
	SPINGO	NA	NA

Each entry in the table shows the F-scores for a classifier (i.e., rows) based on all the OTU sequences in the msd16s dataset, as described in the main text. Two confidence score thresholds (CST), 0.8 and 0.5, were applied for BLCA, RDP Classifier, and SPINGO, the thresholds as in Table 1. Note that the SPINGO program does not produce family-level classification. In addition, Kraken and MEGAN do not provide any probabilistic-based parameters for evaluating the assigned taxa, thus we used their default taxonomic assignments for comparison

## Discussion

Despite the importance of species-level classification, the existing tools either do not classify 16S sequences at the species level or their taxonomic assignments are not reliable. As discussed above, k-mer based methods are intrinsically less accurate than an alignment-based sequence similarity measurement. The k-mer based approaches may be sufficient for high level taxonomic classification, since sequences from different higher taxonomic levels tend to be very divergent. For lower level taxonomic classification, however, particularly species-level classification, we have shown that BLCA significantly outperforms k-mer based methods (e.g., SPINGO, the RDP Classifier, and Kraken) in classification accuracy.

In addition, the Bayesian posterior probability of BLCA quantitatively measures the difference between the best database hit and other database hits, and the bootstrapping principle, adopted by BLCA for providing confidence score, has solid statistical foundation for measuring prediction errors [22]. In this study, we have applied 0.5 and 0.8 as thresholds for the BLCA confidence scores for comparison with other software. The confidence score of BLCA is comparable to that of the RDP Classifier and SPINGO. There is no perfect universal threshold that is suitable for all datasets. We recommend that users consider exploring several different thresholds (e.g., 0.6 and 0.8) to examine if their results are consistent under different thresholds. If not, the users need to be wary that their results may be too sensitive based on the particular threshold they have chosen.

It is worth mentioning that BLCA does not require a training process for classification, which can be more convenient for some users when compared to some other software. For example, the 16S Classifier trains a standard machine-learning model, a Random Forest, with k-mer nucleotides from different regions of 16S rRNA genes. We could not even test our V1–V3, V3–V5, and V6–V9 datasets with the 16S Classifier because the published software has not been trained for this region, even though these regions are widely used in microbiome studies. In contrast, our BLCA program requires no training process at all since our algorithm is based on the alignment between query and reference database sequences. Therefore, users only need to download reference 16S database sequences for BLCA and this allows our method to be easily applied to any other DNA marker gene families for taxonomic classification (e.g., rpoB or 18S rRNA gene sequences). The accompanying BLCA package includes instructions on how to replace the default 16S sequences with the user’s own customized gene family sequences. For example, to demonstrate the flexibility of alternative database sequences, BLCA provides an option to use the Greengenes 16S database and its associated taxonomy [11] instead of the default NCBI 16S database since many researchers may prefer the Greengenes taxonomy.

We have shown that BLCA has significantly higher accuracy than existing taxonomic classification methods at the species level. This higher accuracy comes with the cost of longer computation time. BLCA is not designed for performing taxonomic classification for raw 16S sequences. Instead, raw 16S sequences should be first clustered into OTUs to eliminate redundant or highly similar sequences before performing taxonomic classification, which is a standard procedure for 16S sequence processing by widely used software packages, e.g., QIIME. With 100,000 OTUs, BLCA can have a run-time of approximately 4 days, which is not unusual for modern-day bioinformatics tasks with large datasets. Considering the significant gains in accuracy with our method, we believe that many researchers will find the time tradeoff to be reasonable. In addition, users can divide the input sequences into multiple files and execute BLCA in parallel on computer clusters to hasten the classification process, if necessary. In addition, not all OTUs require species-level classification in practice. Typically, researchers are only interested in a small subset of OTUs, e.g., a list of OTUs that are differentially abundant in different ecosystems (similar to how molecular biologists are often only interested in detailed gene annotations for a small list of differentially expressed genes instead of all of the genes in an organism). In these cases, BLCA may take only a few minutes to classify a subset of several hundreds of OTUs of interest.

## Conclusion

In summary, we have developed a novel computational method that significantly outperforms previously published software for species-level classification accuracy. Its probabilistic-based confidence score helps users evaluate the confidence of the resulting taxonomic assignments based on multiple database hits. In addition, our methods do not require any training, which makes it easily applicable for different regions of 16S rRNA gene or even different phylogenetic marker genes. Despite its higher computational costs, our method is still suitable for large-scale microbiome datasets, providing a valuable alternative option for microbiome researchers who prefer higher classification accuracy.

## Abbreviations

FN: False negative
FP: False positive
LCA: Lowest Common Ancestor
OTU: Operational taxonomic unit
rRNA: ribosomal RNA
TN: True negative
TP: True positive
